# Hope for the best, but prepare for the worst – Diagnostic accuracy of the American College of Surgeons National Surgical Quality Improvement Program – Risk model for patients undergoing abdominoplasty after massive weight loss – Results from a Retrospective Cohort Study

**DOI:** 10.1016/j.jpra.2024.12.002

**Published:** 2024-12-10

**Authors:** Torsten Schulz, Toralf Kirsten, Stefan Langer, Rima Nuwayhid

**Affiliations:** a Department of Orthopaedic, Trauma and Plastic Surgery, University Hospital Leipzig, 04103 Leipzig, Germany; b Medical Informatics Center - Department of Medical Data Science, University Hospital Leipzig, 04103, Leipzig 04103, Germany

**Keywords:** ACS-NSQIP risk calculator, Abdominoplasty, Risk stratification, Massive weight loss

## Abstract

**Background:**

This study aimed to validate the American College of Surgeons (ACS) National Surgical Quality Improvement Program (NSQIP) risk calculator for predicting outcomes in patients undergoing abdominoplasty after massive weight loss.

**Methods:**

Patients’ characteristics, pre-existing comorbidities and adverse outcomes in our department from 2013 to 2023 were collected retrospectively. Adverse events were defined according to ACS-NSQIP standards and predicted risks were calculated manually using the ACS-NSQIP risk calculator. Binary logistic regression and the Brier score were used to assess the diagnostic accuracy of the model.

**Results:**

Among the 337 individuals who underwent abdominoplasty, 251 had achieved significant weight loss before surgery. After excluding 46 cases due to incomplete data, 205 cases remained for analysis. There were 20% cases of serious complications, 26.3% of some complications, 10.2% of readmissions, 18.8% returned to the operating theatre, 15.6% of surgical site infections and 0.5% each of pneumonia and venous thromboembolism. Although the calculator predicted a 1.5% discharge rate to nursing or rehabilitation facilities and a 0.1% rate of sepsis, neither outcome was observed. Elevated American Society of Anesthesiologists (ASA) status was significantly associated with a higher complication rate, except for surgical site infections (SSI) (*p* = 0.06). Additionally, an elevated Body Mass Index (BMI) before post-bariatric surgery and a higher resection weight were both associated with increased rates of return to the operating theatre (*p* = 0.01) and serious complications (*p* = 0.01). Predicted complication rates (0.1%-8.6%) underestimated actual complication rates (0.5%-26.3%). The Brier scores did not differ significantly from the null model for any outcomes except for general complications (*p* = 0.001) and logistic regression models demonstrated low sensitivity (0.0-9.8%) and weak odds ratios (1.28-1.46), indicating limited reliability.

**Conclusion:**

The ACS-NSQIP risk calculator does not reliably predict adverse outcomes in this patient cohort.

## Introduction

Abdominoplasty is a surgical procedure that reduces excess skin and subcutaneous fat. It enhances the aesthetic appearance and alleviates functional limitations in postpartum patients and in cases of cutis laxa due to ageing and other conditions. The rising prevalence of obesity has led to a global increase in both bariatric and post-bariatric surgeries.^1^^,^^2^ Abdominoplasty is one of the most important procedures used to reshape the body after losing weight.^2^ In patients who have experienced massive weight loss, abdominoplasty procedures carry an increased risk of complications due to factors such as a greater amount of excess tissue, higher Body Mass Index (BMI) and potential malnutrition following bariatric surgery.^3^^,^^4^ The incidence of post-bariatric body contouring surgical complications is around 31.5%.^3^ The National Surgical Quality Improvement Program (NSQIP) risk calculator from the American College of Surgeons (ACS) enables physicians to predict the likelihood of adverse events, ranging from surgical site infections (SSI) to mortality, across various types of surgeries.^5^^,^^6^ However, it has not been validated for patients undergoing abdominoplasty after weight loss. The tool's usefulness was tested in a cohort of patients who underwent panniculectomies, showing that the ACS-NSQIP risk calculator was accurate in predicting some, but not all, serious complications.^7^ Characteristics of patients requiring panniculectomy differ from those undergoing abdominoplasty, as does the operative technique, which limits the generalizability of findings to the abdominoplasty cohort. While predictive factors for complications have been studied, a reproducible tool similar to the ACS-NSQIP is still needed. Therefore, the primary purpose of this study was to investigate the applicability of the preoperative NSQIP risk calculator for patients undergoing abdominoplasty after massive weight loss.

## Material and methods

### Data acquisition

The investigation was authorised by the institutional review board as a retrospective analysis of all surgical patients who underwent a procedure with Current Procedural Terminology code 15847 at Leipzig University Hospital from 1 January 2013 to 31 December 2023. The study adhered to the strengthening the reporting of observational studies in epidemiology guidelines. For each abdominoplasty performed at our clinic, data were retrieved from digital patient records. Patient data were prepared with the support of our university hospital's Data Integration Centre, funded by the German Federal Ministry of Education and Research (Grant No. 01KX2121). All post-bariatric operations were registered anonymously through a database query, with cases included based on primary diagnosis codes. Patients treated in other departments, those without massive weight loss and those with incomplete datasets were excluded. According to the UK Commissioning Guide, massive weight loss was defined as a reduction of 50% or more in excess body weight. Excess body weight was calculated as the difference between the patient's BMI and a maximum normal BMI of 25 kg/m².^8^ The collected data were compiled by the Department of Medical Data Science of the Medical Informatics Centre using the statistical software R (RStudio Team 2020, RStudio: Integrated Development Environment for R. RStudio, PBC, Boston, MA, USA). Data obtained from the computerised medical records included demographics (such as age, gender, BMI, laboratory data and anaesthesiology parameters), medical comorbidities and postoperative complications. Similarly, details of the operation performed, such as cut-to-stitch time, liposuction, hernia repair and rectus plication were documented.

### Surgical procedure

The surgical technique involves making an incision that follows the natural curve of the lower abdomen. A flap of skin and fat is elevated cranially up to the xiphoid process. To minimise seroma formation, approximately 5 mm of subcutaneous adipose tissue is preserved on the fascia during preparation and the use of electrocautery is kept to a minimum. The umbilicus, along with a cuff of adipose tissue to ensure perfusion, is prepared and secured to the fascia. To identify and mark the excess tissue, the patient's torso is elevated. After resection, the remaining flap is sutured to the distal incision without tension, to prevent wound complications such as dehiscence or hypertrophic scar formation. The umbilicus is repositioned on the abdominal wall through an inverted V-shaped incision and anchored to the fascia with PDS 3-0 sutures. Two 12 and two 14 Charrière drains are placed in the wound bed. Scarpa's fascia is closed with Monocryl 0, followed by the dermis with Monocryl 3-0 and the skin with continuous intradermal Monocryl 4-0 sutures. The umbilicus is stitched to the dermis in its new position with Monocryl 3-0 and Ethilon 4-0 and then covered with antiseptic dressing, Betadine ointment, fat gauze and adhesive tape. The lower abdominal incision is covered with Steri-Strips. Additional procedures such as liposuction, hernia repair or rectus muscle tightening can be combined with the primary procedure. Patients are advised to remain in a semi-seated position with hip flexion for 7 days postoperatively to reduce tension on the wound.

### Outcome parameters

The calculator estimates the risk for 13 outcomes as defined by the ACS-NSQIP risk score, including serious complications, readmission, return to the operating theatre, surgical site infection, pneumonia, venous thromboembolism, cardiac complications, urinary tract infection, renal failure, sepsis, death and discharge to nursing or rehabilitation facilities within the first 30 days post-surgery. Detailed definitions of these outcome parameters are provided in Supplementary Table 1.

### Statistical analysis

For demographic characteristics and other factors, means and proportions were calculated. Patients’ comorbidities were individually scored manually using the ACS-NSQIP risk calculator (see https://riskcalculator.facs.org/RiskCalculator/) to generate a preoperative risk score. This ACS-NSQIP risk prediction score was validated through calibration and concordance statistics. Calibration was assessed by calculating Brier scores for both a null model and the NSQIP score. The Brier score, determined by the mean squared difference between each patient's predicted probability and the actual outcome, reflects how closely patients’ observed risk aligns with their predicted risk. Both Brier scores were compared for significant differences using a two-tailed t-test. Concordance was evaluated using logistic regression and the Hosmer-Lemeshow goodness-of-fit test. These logistic regression models were characterised by their coefficients, corresponding odds ratios, sensitivity and specificity. The predictive power was visualised by constructing receiver operating characteristic (ROC) curves and calculating the area under the curve (AUC). A ROC curve evaluates a model's ability to distinguish between higher and lower-risk patients, while the AUC, which ranges from 0.5 (chance) to 1 (perfect), indicates the model's accuracy in correctly classifying cases as either with or without a disease. Statistical analyses were performed using IBM SPSS Statistics, Version 29 (IBM Corporation, Armonk, NY, USA). To reject the null hypothesis and accept the alternative hypothesis, a significance level of less than 0.05 was set.

## Results

### Patient characteristics

**Figure 1:**
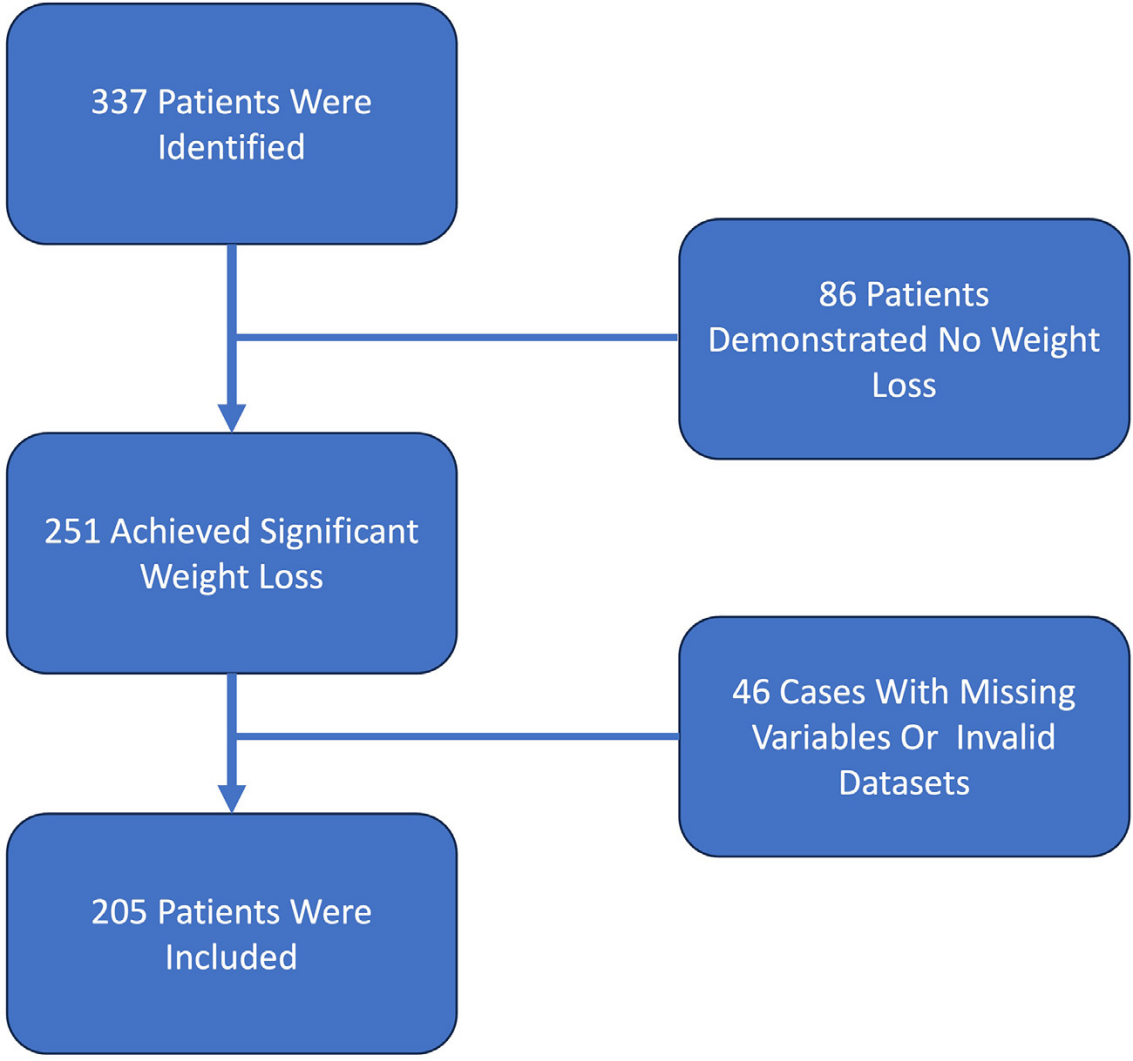
Flow chart and study design.

A total of 337 patients underwent abdominoplasty at our clinic between January 2013 and December 2023. Of these, 251 had previously achieved significant weight loss. Forty-six patients were excluded due to invalid datasets or missing variables, leaving 205 patients (Figure 1). The 205 participants were categorised as follows: those with no complications (n = 151), any complication (n = 54), serious complications (n = 41), readmission (n = 21), return to the operating theatre (n = 39), surgical site infection (n = 32), pneumonia (n = 1) and venous thromboembolism (n = 1). Adverse events such as cardiac complications, urinary tract infections, renal failure, sepsis, death and discharge to nursing or rehabilitation facilities were not observed within the first 30 days post-operation. Further analysis of pneumonia and venous thromboembolism was not feasible due to the limited number of events (1).

The mean age of the cohort was 46.8 years (Table 1). Over two-thirds of each group were female, with most having lost weight through bariatric procedures. The average bariatric patient presented with an initial BMI of 50.0 kg/m², achieving a relative reduction of 19.3 kg/m² and a preoperative BMI of 30.7 kg/m². The interval between bariatric and post-bariatric surgery was 1194.9 days. Most patients were classified as ASA II, with smaller percentages in ASA III and I. Additional procedures included liposuction in 19.3%, hernia repair in 12.1% and rectus plication in 18.8% of cases. The average cut-to-stitch time was 144.6 minutes, with an average resection weight of 2815.5 grams and the mean hospital stay was 6.5 days. Hypertension, hypothyroidism, smoking, diabetes mellitus type II and venous insufficiency were the most common pre-existing comorbidities.

**Table 1:** Patient baseline data.

	Total Population (n = 205)
Male, N/%	56/ 27.1 %
Female, N/%	149/ 72.0 %
Age in years, Mean ± SD	46.8 ± 11.3

Bariatric data	
post-bariatric procedures, N/%	150/ 72.5%
Weight loss by diet, N/%	55/ 26.6%
BMI, kg/m^2^_initial,_ Mean ± SD	50.0 ± 9.8
BMI, kg/m^2^_after weight loss_, Mean ± SD	30.7 ± 5.1
BMI, kg/m^2^_Delta_, Mean ± SD	19.3 ± 8.8
Time between bariatric and post-bariatric surgery in days, Mean ± SD	1194.9 ± 545.1

Perioperative metrics	
ASA I/II/III, N/%	23/159/23, 11.1/76.8/11.1%
Liposuction, N/%	40/19.3%
Rectus plication, N/%	39/18.8%
Hernia closure by direct suture, N/%	25/12.1%
Cut-to-stitch time, minutes, Mean ± SD	144.6 ± 37.7
Resection weight, gram, Mean ± SD	2815.5 ± 1710.7
Hospital stay, days, Mean ± SD	6.5 ± 2.7
Hypertension	97/ 46.9 %
Hypothyroidism	63/ 30.4 %
Smokers	50/ 24.2 %
Diabetes mellitus type II	39/ 18.8 %
Venous insufficiency	37/ 17.9 %

An increased ASA status was significantly associated with any complications (p = 0.01), serious complications (p = 0.006), readmissions (p = 0.04) and returns to the operating theatre (p < 0.001) (Table 2). Resection weight was significantly higher in patients with any complications (p = 0.02), serious complications (p = 0.01) or in those who returned to the operating theatre (p = 0.01), suggesting a possible link to increased complication rates. An elevated BMI before post-bariatric surgery also correlated with higher rates of returns to the operating theatre (p = 0.01) and serious complications (p = 0.01) (Table 2). Patient age, cut-to-stitch time and the interval between bariatric surgery and abdominoplasty showed no significant differences between groups (Supplementary Table 2).

**Table 2:** Comparison of patient's risk factors in dependence of the event categories.

	Any Complication			Serious Complication			Readmission			Return to the Operating Theatre			Surgical Site Infection		
	(n = 151)	(n = 54)	*P*	(n = 164)	(n = 41)	*P*	(n = 184)	(n = 21)	*P*	(n = 166)	(n = 39)	*P*	(n = 151)	(n = 32)	*P*
ASA Status, Mean ± SD	1.9 ± 0.4	2.1 ± 0.5	**0.015**	1.9 ± 0.4	2.2 ± 0.5	**0.006**	1.9 ± 0.4	2.2 ± 0.5	**0.04**	1.9 ± 0.4	2.2 ± 0.5	**<0.001**	1.9 ± 0.4	2.1 ± 0.5	0.06
BMI, kg/m^2^_initial_ Mean ± SD	49.7 ± 9.7	50.9 ± 10.2	0.45	49.8 ± 9.6	50.9 ± 10.6	0.52	50.0 ± 10.0	50.1 ± 7.6	0.96	49.7 ± 9.6	51.3 ± 10.6	0.37	49.9 ± 10.1	50.4 ± 8.1	0.77
BMI, kg/m^2^_reduction_ Mean ± SD	19.4 ± 8.8	19.2 ± 9.1	0.89	19.5 ± 8.7	18.4 ± 9.3	0.48	19.6 ± 9.0	17.0 ± 6.4	0.11	19.4 ± 8.7	18.7 ± 9.4	0.66	19.4 ± 9.1	18.7 ± 7.3	0.64
BMI, kg/m^2^_after massive weight loss_ Mean ± SD	30.3 ± 4.9	31.7 ± 5.5	0.10	30.2 ± 4.9	21.5 ± 5.3	**0.01**	30.4 ± 5.0	33.0 ± 5.5	0.05	30.2 ± 4.9	32.6 ± 5.5	**0.01**	30.5 ± 4.9	31.6 ± 5.9	0.31
Resection weight in gram Mean ± SD	2630.9 ± 1626.1	3320 ± 1847.6	**0.02**	2649.8 ± 1658.8	3447.7 ± 1779.9	**0.01**	2730.7 ± 1688.8	3547.7 ± 1769.0	0.06	2651.9 ± 1648.5	3483.7 ± 1819.2	**0.01**	2710.6 ± 1668.6	3372.5 ± 1850	0.08

### Outcome parameters

There was a significant difference between the predicted risks, calculated by entering individual patient characteristics into the ACS-NSQIP online risk calculator and the observed risks in the categories of serious complications, any complications, readmissions, return to the operating theatre and SSI. One patient developed pneumonia following surgery and another patient experienced a thromboembolic event. There were no actual occurrences of cardiac complications, urinary tract infections, renal failure or death, despite low predicted risks. Additionally, although discharge to a nursing or rehabilitation facility was predicted in 1.5% and sepsis in 0.1% of cases, neither occurred. Consequently, further calculation of prediction probabilities for these categories could not be conducted. The Brier scores for the ACS-NSQIP calculator were not statistically significantly different from the null Brier score for any of the outcomes, except for any complications (p = 0.001) (Table 3).

**Table 3:** Estimated risk model provided by the ACS-NSQIP.

30 Day Outcomes	Predicted Risk	Actual Risk	Brier_ACS-NSQIP_	Brier_0-Model_	*P*
Serious Complication (n = 41)	4.5 %	20.0 %	0.21	0.21	0.81
Any Complication (n = 54)	8.6 %	26.3 %	0.75	0.78	0.001
Readmission (n = 21)	3.1 %	10.2 %	0.11	0.11	0.97
Return to Operating Theatre (n = 39)	1.6 %	18.8 %	0.02	0.02	0.29
Surgical Site Infection (n = 32)	5.2 %	15.6 %	0.28	0.28	0.91

**Figure 2:**
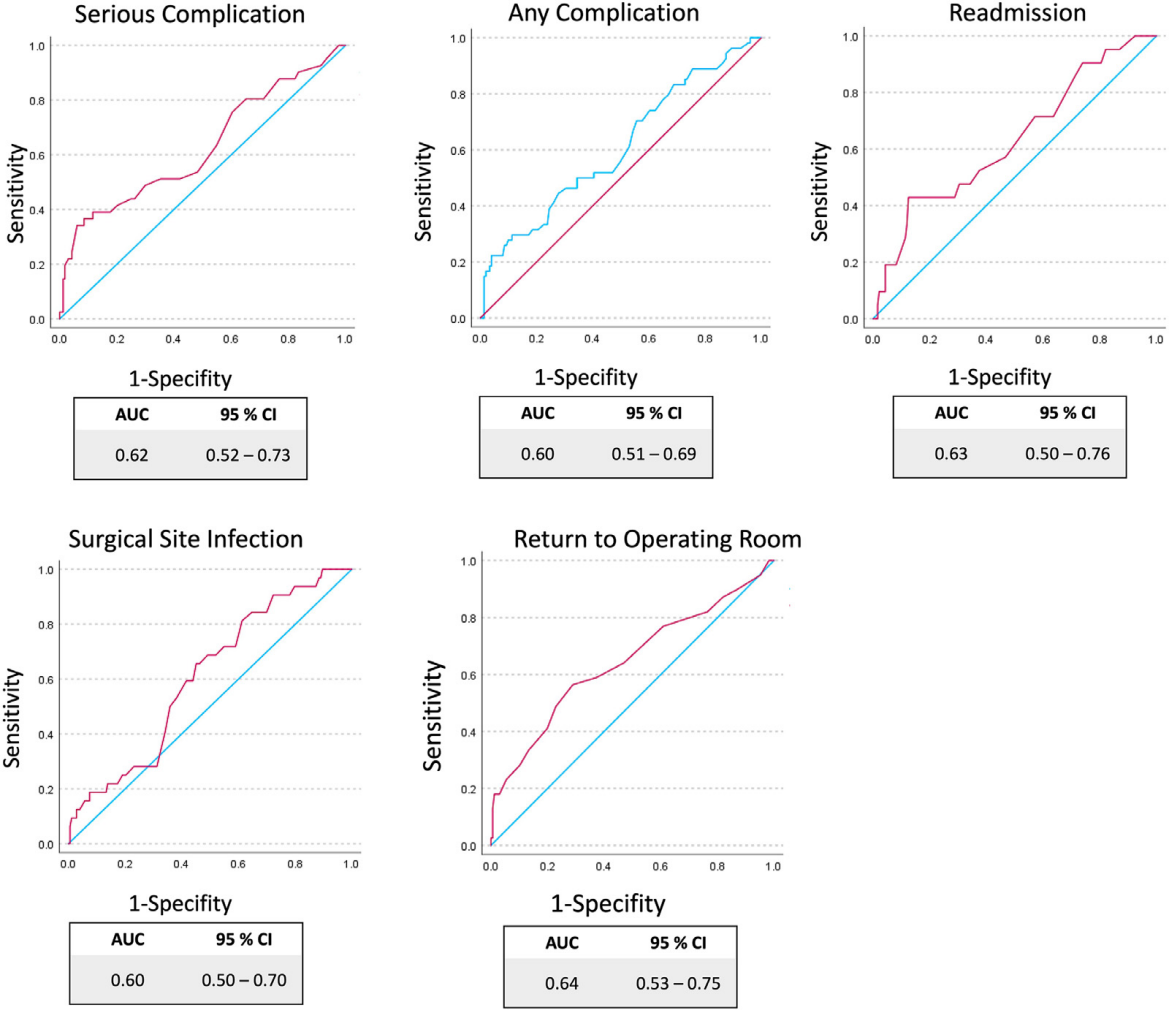
Predictive power of the model described by the receiver operating curves and the area under the curve for each category of the ACS NSQIP Risk Calculator.

The logistic regression models fit the data demonstrated by the Hosmer-Lemeshow test results (p < 0.05) in the categories of serious complications, any complications, readmissions, return to the operating theatre and SSI (Table 4). However, the models struggle with low sensitivity and perform well in identifying negative cases characterised by high specificity. The odds ratios and the corresponding coefficients were statistically significant but weak, with the exception of the category ‘return to the operating theatre’, which had an odds ratio of 4. The ROC curves demonstrate a fair ability to predict various outcomes, as indicated by the AUC values between 0.60 and 0.64 (Figure 2). The wide confidence intervals suggest considerable uncertainty in the model's predictions.

**Table 4:** Binary Logistic Regression Model provided by the ACS-NSQIP.

30 Day Outcomes	Hosmer - Lemeshow Test / P	Sensitivity in %	Specificity In %	Coefficients	*P*	Odd s Ratio (95 % CI)
Serious Complication (n = 41)	0.168	9.8	98.8	0.382	<0.001	1.46 (1.18-1.81)
Any Complication (n = 54)	0.102	5.6	98.7	0.180	0.05	1.19 (1.05-1.35)
Readmission (n = 21)	0.359	0.0	100.0	0.260	0.04	1.29 (1.01-1.66)
Return to Operating Theatre (n = 39)	0.596	5.1	99.4	1.386	<0.001	4.00 (1.77-9.03)
Surgical Site Infection (n = 32)	0.781	0.0	99.4	0.249	0.03	1.28 (1.02-1.61)

## Discussion

In this trial, the validity of the ACS-NSQIP calculator for abdominoplasties in patients with massive weight loss was examined for the first time. An appropriate risk-benefit assessment and thorough patient education are essential. The ACS-NSQIP was introduced in 2013 to evaluate risks based on individual patient characteristics. This tool is grounded in reliable clinical data from multiple institutions and is designed to estimate the risks associated with most surgical procedures.^5^ Our results showed that the risk calculator systematically underestimates risks in the predefined categories: any complication, serious complication, readmission, return to the operating theatre and surgical site infection. All other categories were predicted but did not actually occur, indicating an overestimation by the surgical risk calculator. Therefore, the reliability of the model appears to be weak for this specific procedure and framework conditions. One possible reason for this inaccuracy is that known risk factors identified in the literature, such as a history of massive weight loss due to bariatric surgery, are not included in the calculator's queries.^3^ Moreover, Suresh and colleagues demonstrated similar results for 264 patients who underwent panniculectomy. The authors concluded that the overall risk of complications in this particular group of patients is underestimated.^6^ Further, Basta et al. demonstrated that the ACS surgical risk calculator significantly underestimated the incidence of SSIs, serious complications and length of stay following open ventral hernia repair in a cohort of 142 patients.^7^ A recent meta-analysis also published by Basta and colleagues summarised, that the ACS risk calculator has repeatedly proven to be a poor discriminator of complications in the field of plastic surgery.^9^ Cohen et al. examined potential ways for enhancement of the model, demonstrating that recalibration may be necessary as the model provided inaccurate estimates for patients in both the lowest and highest risk classifications.^5^

Moreover, other publications on the external validity of the risk calculator revealed a very heterogeneous picture. Some authors claimed a reliable prediction of mortality in patients undergoing emergency abdominal surgery.^10^ Especially in lower-risk groups, the ACS-NSQIP risk calculator was able to predict the postoperative mortality.^11^ However, the ACS-NSQIP surgical stratification showed a poor individual risk prediction for all outcomes in geriatric patients with colorectal cancer.^12^ Other publications in colorectal surgery painted a similar picture.^13^ In spine surgery, the risk calculator seemed to be an adequate predictive tool for a subset of complications after Anterior Lumbar Interbody Fusion, including acute kidney injury/progressive renal insufficiency, surgical site infection and discharge to non-hospital facilities. Nevertheless, the ACS-NSQIP calculator demonstrated limited validity for all other categories of the risk model.^14^ The calculator seems to be able to predict a total number of complications, but not individual risks.^15^ These data were consistent with a recently published meta-analysis, which concluded that the ACS-NSQIP risk calculator is an ineffective instrument for predicting outcomes in head and neck surgery.^16^ In addition, for endovascular and open aortic aneurysm repair, the ACS risk calculator was found to be inaccurate.^17^ In the context of orthopaedic oncological surgery regarding distal femur arthroplasty the ACS-NSQIP did not adequately predict the incidence of complications.^18^

It should be noted, that this work also demonstrates limitations. Because only one case of pneumonia and one thromboembolic event were observed and no occurrences of cardiac complications, urinary tract infections, renal failure, sepsis, death or discharge to a nursing or rehabilitation facility was found, no further statements regarding statistical predictive accuracy are possible. Because of its single-centre design, there is a possibility that our findings may be influenced by what could be regarded as a ‘local treatment effect’.^19^ The local treatment effect refers to the impact of an intervention or treatment on a specific subgroup or localised portion of a population, rather than on the entire population or a broader group. Therefore, caution is always advised when interpreting our results, as external validity is not always automatically guaranteed. There are numerous potential, unmentioned confounders that may vary from clinic to clinic and from surgeon to surgeon. For instance, surgeon-related confounders include a lack of atraumatic dissection techniques, excessive use of electrocautery, tensioned wound closure and inadequate postoperative care, such as improper staged bed positioning, inappropriate compression or incorrect mobilisation, all of which could influence the complication rate.^2^

Ultimately, the question remains as to why there was a significant difference between the predicted and actual complications in our study, and why the ACS-NSQIP model did not accurately capture this. In the literature, a preoperative BMI > 30, increased intraoperative resection weight and a history of bariatric surgery have been identified as risk factors in body contouring procedures following massive weight loss.^3^^,^^20^ While BMI is recorded preoperatively by the risk calculator, resection weight cannot be objectively estimated before surgery, making its inclusion in future risk calculators impractical. Furthermore, the risk calculator does not differentiate between patients with a history of bariatric surgery and those undergoing the procedure for aesthetic reasons. Despite these limitations, there remains a 60-87% increased risk of complications in the post-bariatric group compared to non-bariatric patients.^20^ Based on the literature and our findings, we recommend that the ACS-NSQIP include an inquiry regarding a history of bariatric surgery to enhance its future predictive accuracy. Other potential factors could include patients’ nutritional status, indicated by laboratory markers of malnutrition, such as serum albumin levels or vitamin deficiencies, which can negatively impact wound healing.^21^^,^^22^ A limitation of our study is the retrospective design of this study. It is possible that inaccuracies or missing data within the electronic medical record may have introduced confounding factors into the observed results. Nonetheless, this underscores the need for establishing a national database specific to the field of plastic and reconstructive surgery. Such a database would enable a more comprehensive understanding of the factors that should be considered when assessing postsurgical risk in this patient population. Future research should focus on risk assessment and the external validity of abdominoplasty in patients without massive weight loss. Additionally, the ACS-NSQIP risk calculator for body contouring procedures should be revised to include well-established risk factors, such as a history of bariatric surgery. Furthermore, it would be desirable for the risk calculator to include other post-bariatric procedures, such as arm or thigh lifts.

## Conclusion

The ACS-NSQIP risk calculator does not accurately predict the likelihood of adverse events of patients undergoing abdominoplasties after massive weight loss. Therefore, the risk calculator cannot be considered a reliable tool in this patient cohort. Well-established risk factors specific to the cohort of post-bariatric patients, such as previous bariatric surgeries, need to be incorporated to improve the model's performance and calibration, making it more reliable for procedure planning and patient education.

## Declaration of competing interest

None.

## Appendix Supplementary materials

Image, application 1
