# Corrigendum to `Hope for the best, but prepare for the worst – Diagnostic accuracy of the American College of Surgeons National Surgical Quality Improvement Program – Risk model for patients undergoing abdominoplasty after massive weight loss – Results from a Retrospective Cohort Study' [JPRAS Open 43 (2025) 347–356]

**DOI:** 10.1016/j.jpra.2026.04.019

**Published:** 2026-04-22

**Authors:** Torsten Schulz, Toralf Kirsten, Katharina Theresa Vogel, Stefan Langer, Rima Nuwayhid

**Affiliations:** a Department of Orthopaedic, Trauma and Plastic Surgery, University Hospital Leipzig, 04103 Leipzig, Germany; b Medical Informatics Center - Department of Medical Data Science, University Hospital Leipzig, Leipzig 04103, Germany

The authors regret that the authorship line in the original publication was incorrect. The corrected authorship and affiliations are presented above.

The authors would like to apologise for any inconvenience caused.

