# Supplementary material for: Hope for the best, but prepare for the worst – Diagnostic accuracy of the American College of Surgeons National Surgical Quality Improvement Program – Risk model for patients undergoing abdominoplasty after massive weight loss – Results from a Retrospective Cohort Study
Source: JPRAS Open. 2024 Dec 10;43:347–56. doi: 10.1016/j.jpra.2024.12.002 (PMC11751431; doi:10.1016/j.jpra.2024.12.002)
Supplement: Supplementary file 1 [file mmc1.docx]

**Supplementary Table 1.** Definition of Endpoints and Critical Conditions

| **Category** | **Definition** |
| --- | --- |
| Primary Endpoint | Occurrence of a serious complication or any complication |
| Serious Complication | Defined by the ACS-NSQIP Risk Score as critical conditions, including cardiac arrest, myocardial infarction, pneumonia, progressive renal insufficiency, acute renal failure, pulmonary embolism, deep vein thrombosis, return to the operating theatre, deep incisional or organ space SSI, systemic sepsis, unplanned intubation, urinary tract infection, and wound disruption |
| Any Complication | Includes all serious complications plus superficial SSI, extended ventilator use (>48 hours), and stroke; covers a range of both severe and less severe issues |
| Readmission | Re-hospitalization within 30 days post-surgery due to adverse events |
| Return to Operating Theatre | Need for a second surgical procedure due to complications |
| Critical Conditions | |
| Cardiac Arrest | Absence of cardiac rhythm or chaotic rhythm requiring CPR |
| Myocardial Infarction | Identified by ECG changes, elevated troponin levels, or physician diagnosis |
| Pneumonia | Lung infection diagnosed via radiologic and clinical criteria |
| Progressive Renal Insufficiency | Increase in creatinine >2 mg/dl without dialysis requirement |
| Acute Renal Failure | Postoperative renal dysfunction requiring dialysis |
| Pulmonary Embolism | New thrombus in the pulmonary venous system, confirmed by imaging, requiring anticoagulation or vena cava intervention |
| Deep Vein Thrombosis | New thrombus in the deep venous system of the legs, requiring anticoagulation |
| Deep SSI | Infection of deep soft tissues of the incision, identified by purulent drainage, dehiscence, abscess, or physician diagnosis |
| Organ Space SSI | Infection involving anatomical areas (e.g., organs) manipulated during surgery, indicated by purulent drainage, positive culture, abscess, or physician diagnosis |
| Systemic Sepsis | Severe systemic infection requiring medical intervention |
| Unplanned Intubation | Unexpected insertion of a breathing tube for ventilation support |
| Urinary Tract Infection | Bladder infection confirmed through clinical symptoms and laboratory tests (urine culture, positive dipstick) or need for antimicrobial therapy |
| Wound Disruption | Separation or opening of a surgical wound |
| Extended Ventilator Use | Requirement for mechanical ventilation for more than 48 hours |
| Stroke | Sudden disruption of blood supply to the brain, resulting in neurological impairment |

|  | **Any Complication** | | | **Serious Complication** | | | | **Readmission** | | | **Return to the Operating Theatre** | | | | **Surgical Site Infection** | | |
| --- | --- | --- | --- | --- | --- | --- | --- | --- | --- | --- | --- | --- | --- | --- | --- | --- | --- |
|  | (n=151) | (n=54) | P | | (n=164) | (n=41) | P | (n=184) | (n=21) | P | (n=166) | (n=39) | P | (n=151) | | (n=32) | P |
| Age in years, Mean ± SD | 47.2 ± 10.9 | 45.4 ± 12.3 | 0.34 | | 46.7 ± 11.1 | 46.8 ± 12.1 | 0.96 | 46.9 ± 11.1 | 45.7 12.9 | 0.68 | 46.7 ± 11.1 | 47.0 ± 12.3 | 0.90 | 47.2 ± 11.0 | | 44.1 ± 12.5 | 0.18 |
| Cut-to-stitch time, minutes, Mean ± SD | 142.9 ± 36.2 | 149.4 ± 41.4 | 0.30 | | 143.7 ± 37.8 | 148 ± 37.4 | 0.50 | 143.6 ± 37.4 | 153.5 ± 39.8 | 0.29 | 143.8 ± 37.7 | 148.0 ± 38.0 | 0.53 | 142.6 ± 36.4 | | 155.4 ± 43.1 | 0.12 |
| Time between bariatric surgery and abdominoplasty in days, Mean ± SD | 1227.8 ± 584.4 | 1101.3 ± 405.5 | 0.14 | | 1216.9 ± 578.3 | 1107.1 ± 380.1 | 0.21 | 1212.65 ± 565.4 | 1046.9 ± 302.5 | 0.25 | 1212.3 ± 578.1 | 1122.6 ± 377.1 | 0.31 | 1222.0 ± 569.7 | | 1059.4 ± 380.8 | 0.08 |

**Supplementary Table 2** Definition of Endpoints and Critical Conditions
